# A novel approach to phase-contrast velocity offset correction by in vivo high-SNR acquisitions

**DOI:** 10.1186/1532-429X-15-S1-P56

**Published:** 2013-01-30

**Authors:** Merlin Fair, Peter D Gatehouse, Andreas Greiser, Peter Drivas, David N Firmin

**Affiliations:** 1 Royal Brompton Hospital, London, UK; 2 Siemens Medical Systems, Erlangen, Germany

## Background

Baseline offset errors on phase-contrast velocity images can be corrected using stationary tissue, for example subtracting fitted corrections from the image (1). Although corrections are often curved over the FOV, 1st order (linear) fitting is typical. This may partly be due to low SNR of static tissue making higher-order fitting unreliable (2). Aim: To evaluate a new method acquiring additional high SNR velocity images specifically to improve offset correction.

## Methods

**Figure 1:**
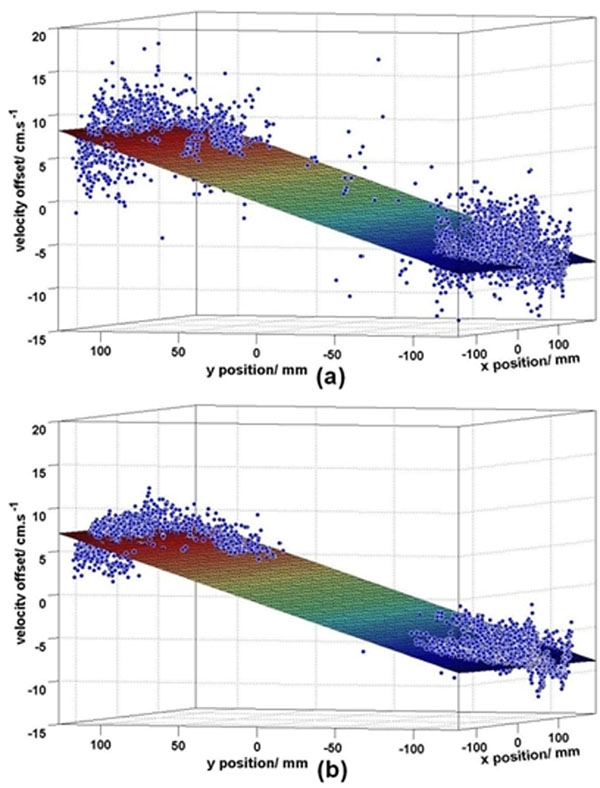
Example of the 1st order surface fits for the original (a) and thicker slice high-SNR (b) pulse sequences, with the stationary tissue shown as blue dots. Improved SNR is clearly visible due to the thicker slice excited by the shorter RF pulse with no sequence timing or gradient alterations so the offset error does not change.

A retrospectively-gated cine phase-contrast sequence was edited so that the RF pulse was 25% of its normal duration, with all other pulse timings and amplitudes unchanged. This excites a thicker slice in the patient, increasing the SNR but without changing the offset error. The modified sequence used an extra breath-hold after the original clinical flow sequence, on 18 vessels in 10 patients. The high-SNR image was not for vessel flow measurement, only baseline correction (Figure 1). Static tissue correction surface fits were applied to all velocity images for a range of polynomial orders (linear to 5th) and static tissue acceptance thresholds (λ in % of all pixels, 5-50 in steps of 5 ) (2). The accuracy of velocity corrections at the position of the vessel of interest was assessed using post-patient phantom scanning (3) as "gold standard".

## Results

**Figure 2:**
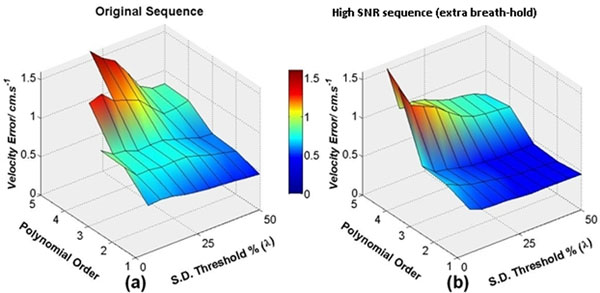
Mean residual velocity error after correction, absolute values averaged across all 18 flow studies as a function of the polynomial order and pixel acceptance % (λ) used for the surface fit, using (a) the original pulse sequence and (b) the new high-SNR pulse sequence acquired aiming for higher fitting accuracy (note: not for flow measurement). Residual errors above 1.8cm/s have been removed from both plots for clarity.

Compared to the clinical scan (Figure 2 left), the high-SNR scan showed small reductions of residual error after correction (Figure 2 right), particularly for higher orders. At best, applying 2nd order fitting to the high SNR image at λ = 50% left a residual error of 0.42±0.35cm/s (n=18, mean[of absolute values] ± stdev) compared with identical fitting parameters on the original flow image 0.56±0.64cm/s. Improvements in first-order fitting accuracy were also small (λ = 25%, high-SNR 0.47±0.33cm/s vs original 0.54±0.44cm/s). Overall, 2nd order fitting on the high SNR image was marginally more accurate than first order fitting on the conventional flow image.

## Conclusions

Discussion & Conclusions: The new high-SNR method improved fitting of higher order surfaces for offset correction, but with marginal improvement of accuracy. For some protocols and scanners with larger uncorrected offsets and/or lower SNR in the clinical flow study, this method might have more impact. The reduced stdev implies that this new method may improve reliability. However, with this data the improved accuracy of correction was insufficient to justify the extra breath-hold with each phase-contrast scan.

## Funding

Cardiovascular Biomedical Research Unit funding from the National Institute for Health Research.
